# Primary cerebral hydatid cyst in a pregnant woman: A case report

**DOI:** 10.1016/j.ijscr.2025.111256

**Published:** 2025-04-04

**Authors:** Ahmad Muhammad, Ahmad Rami Alhayek, Ahmad Abd Alhai Rehawi, Yousef Hanna

**Affiliations:** a Faculty of Medicine, University of Aleppo, Aleppo, Syria; b Department of Neurosurgery, Syrian Arab Republic Ministry of Health, Aleppo, Syria

**Keywords:** Hydatid cyst, Echinococcosis, Brain, Pregnancy, Albendazole, Case report

## Abstract

**Introduction:**

Hydatid cyst disease is a zoonotic parasitic disease. The most common pathogen behind the disease is Echinococcus granulosus. The liver and lungs are the most common sites to occur, while primary intracranial cysts rarely occur.

**Case presentation:**

A 32-year-old female patient in the 17th week of pregnancy presented with a chief complaint of a frontal headache that started a month ago and intensified ten days ago, the patient is living in a rural region and herding sheep. Neurological examination showed slowly reacting pupils, dysarthria, and right-sided ptosis. The investigation was suggestive of a diagnosis of hydatid cysts. Emergent surgery was performed and the cyst was removed. The clinical condition was followed up and she gave birth by cesarean section.

**Discussion:**

The hydatid cyst is a differential diagnosis of any cystic lesion in endemic regions to echinococcosis. MRI is the main investigation. The treatment of cerebral hydatid cysts is both medical and surgical. Surgical treatment should be considered whenever possible. Prompt diagnosis and treatment are crucial, considering that pregnancy can influence the available treatment options.

**Conclusion:**

Here we report a successful treatment of cerebral hydatid cyst during pregnancy. The incidence of cerebral hydatid cysts during pregnancy is extremely low, and there is a lack of documentation on this condition.

## Highlights

• Hydatid cysts are very rare in pregnancy, especially cerebral hydatid cysts.
• The clinical manifestations differ, and MRI is the main investigation.
• Surgery has a key role in treatment.
• Prompt and appropriate diagnosis and treatment are required.

## 1 Introduction

**Fig. 1:**
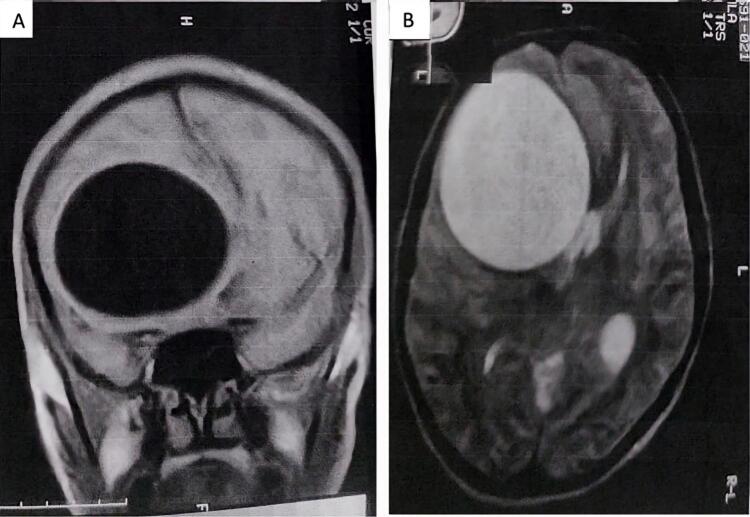
Brain MRI: Rounded well-limited lesion in Hyposignal T1 (A), and Hypersignal T2 (B) with a maximum diameter of 50 mm.

**Fig. 2:**
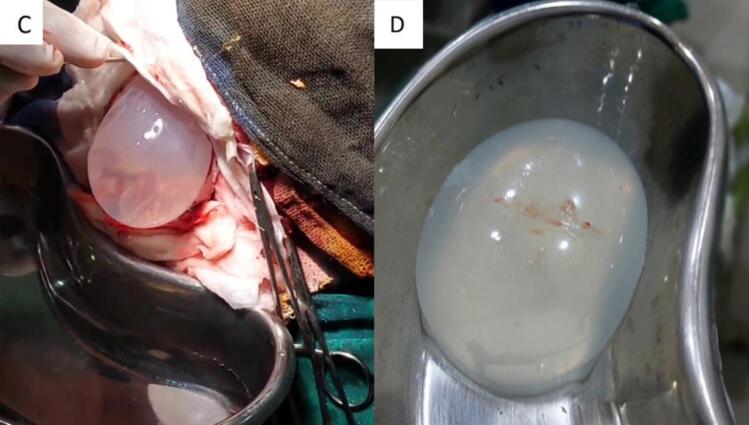
C: Preoperative image, extraction of the cyst, D: after removing the cyst.

Hydatid cyst disease is a zoonotic parasitic disease. The most common pathogen behind the disease is Echinococcus granulosus. Humans are infected due to consumption of contaminated food and water containing the Echinococcus granulosus eggs or close contact with the host like dogs [1]. Larvae emerge from the ingested eggs in the small intestine, then enter the bloodstream after penetrating the intestinal wall, continuing to the liver or lungs, while the remaining 10 % of larvae reach other organs like the brain by entering the systemic circulation [2]. Most often, hydatid cysts are seen in the liver (55–70 %) and lungs (15–35 %), whereas primary intracranial cysts are rare, accounting for only 1.7 % of cases [2]. The disease is poorly understood in pregnant women due to the low incidence during pregnancy which ranges from 1 in 20,000 to 30,000 [1]. (See Fig. 1, Fig. 2.)

This case report has been reported in line with the SCARE Criteria [3].

## 2 Presentation of case

A 32-year-old female patient in the 17th week of pregnancy presented to our emergency department with a chief complaint of a frontal headache that began a month prior and intensified over the past ten days, as well as drowsiness, sphincter incontinence, and without vomiting, fever, or convulsions. The patient lives in a rural region and herds sheep. At admission, her Glasgow coma scale was 14/15. Neurological examination showed slowly reacting pupils, dysarthria, and right-sided ptosis. The obstetrical exam was normal.

An MRI brain was performed, revealing a large cystic lesion in the right frontal lobe with a diameter of 50 mm, putting pressure on the right lateral ventricle, leading to initial stages of subfalcine herniation. The described lesion was isointense to cerebrospinal fluid on Fluid-attenuated inversion recovery (FLAIR) sequences, which indicated a hydatid cyst. The epidemiological context, including sheep grazing and rural living, further supported this diagnosis. The chest X-ray and abdominal ultrasound were normal.

Emergent surgery was performed, and under general anesthesia a wide frontoparietal craniotomy was performed and the dura was dissected. Hydropulsion with hypertonic saline was applied between the parenchyma and the cyst according to the Arana-Iniguez technique, followed by a warm saline irrigation to facilitate separation. The cyst was then allowed to fall out by lowering the head of the operating table. Careful handling was maintained throughout all operative steps to avoid rupturing the cyst. The cyst was successfully removed without incident. Albendazole was administered post-operatively and again post-birth to decrease the risk of recurrence.

The clinical condition was followed up, and the patient was discharged after five days in good condition, with improvement in clinical symptoms. She gave birth to a healthy baby girl who was born by cesarean section without incident.

## 3 Discussion

The cerebral hydration cyst, or cerebral hydatidosis, is an intracerebral parasitic lesion that develops from Echinococcus Granulosus. The dog is the main definitive host of Echinococcus Granulosus, whereas the human is an accidental host [4]. Typically, cerebral hydatidosis manifests as solitary cysts in the primary type, and multiple cysts in the secondary type.

Cerebral hydatidosis can arise anywhere in the brain, but it typically arises intra-parenchyma and in rare cases extradural, or a combination of these localizations. Cysts are usually supratentorially localized in the distribution of the terminal branches of the middle cerebral artery, usually temporal-parietal-occipital [6].

The brain is a rare place for hydatidosis, and the clinical manifestations differ, with developing intracranial pressure increase syndrome (IPIS), and Neurological signs may appear or be absent [4]. The most common symptoms are Headache and vomiting, which occur as a result of increased intracranial pressure. Other findings in clinical examination are deteriorating consciousness, loss of strength, papilledema, and pathological reflexes [2]. Vomiting during pregnancy can be misleading when considering the hypothesis of intracranial hypertension syndrome. This must be thoroughly investigated to find other elements of this syndrome and focal neurological signs [4]. It takes a high degree of suspicion to diagnose a hydatid cyst. In endemic regions to echinococcosis, the hydatid cyst is a differential diagnosis of any cystic lesion [1]. It is suggested that the decrease in cell-mediated immunity plays a role in promoting rapid cyst growth, leading to potential significant enlargement in size and the onset of symptoms and complications [1].

MRI is the main investigation, showing a lesion in hypo-signal T1, and hyper-signal T2, and it is more effective in identifying multiple cerebral hydatid cysts. It helps surgical planning by precisely defining the lesion anatomy of the surrounding structures. But in the case of calcified cysts, CT is a superior option [4].

In comparison to other localizations, the contribution of hydatid serology is still minimal. It can be used in diagnosis and post-operatively after considering its low sensitivity. The histology diagnostic shows the cyst's germinative membrane [4].

Surgery has a key role in treatment, aiming to remove the cyst completely without rupture, and it should be implemented whenever possible. Many different cyst removal techniques have been proposed, which all share in atraumatic techniques to prevent cyst rupture [4,7]. The Dowling technique, which Arana-Iniguez and San Julian later modified, is a widely used surgical method to treat central nervous system hydatid cysts [7]. This technique can be implemented easily, allowing the cyst to be removed without rupture and with the least harm to the cerebral tissues. Another technique is aspiration puncture, which is used in case of cysts with a high risk for rupture, such as cysts of the fourth ventricle, brain stem, and thalamus, so it is less usually used [4].

Systemic antiparasitic treatment (Albendazole) efficiently treated liver and lung hydatid cysts and recently, the brain [2]. The administrated dose is 15 mg/kg/day for 3 months, and small doses may be used for treating hydatidosis during pregnancy [2,4]. Even if Albendazole's teratogenic effects on animals are not proven in humans, it should not be administrated during the first trimester, and it is approved that Albendazole belongs to the C category for use in pregnancy. In case the benefit for the patient outweighs the possible harm to the fetus, the physician May administer the drug. After the fetus organogenesis is done, administrating Albendazole is safer [2].

To the best of our knowledge, only two cases have been documented previously, with this being the third. Our case is notable and distinguished because unlike the previous cases, which were diagnosed during the third trimester of pregnancy, our case was diagnosed during the second trimester. Additionally, it is noteworthy that symptoms began to develop from the end of the first trimester and persisted into the beginning of the second trimester, lasting for about a month, which is relatively fast, aligning with the proposed role of decreased cell-mediated immunity during pregnancy. Furthermore, Albendazole was administered post-operatively, as the patient was in her second trimester, a stage during which the drug is considered safer. This case emphasizes the impact of pregnancy on the development and treatment of the disease, a topic with little data that needs more research.

The prognosis is good for mother and fetus if the condition is identified quickly and treated to achieve the best possible outcomes. One method of preventing this is controlled sheep culling [4].

## 4 Conclusion

Intracranial hydatid disease in pregnancy is an exceptionally rare clinical condition. Diagnosing a hydatid cyst requires a high degree of suspicion, particularly in endemic regions where it is a critical differential diagnosis for cystic lesions. Timely diagnosis and appropriate intervention are crucial to optimize outcomes for both the mother and the fetus. Surgical treatment remains the cornerstone of management whenever feasible. The administration of Albendazole necessitates careful evaluation of potential risks to the fetus.

## Consent for publication

Written informed consent was obtained from the patient to publish this case report and accompanying images. A copy of the written consent form is available for review by the editor-in-chief of this journal upon request.

## Ethical approval

Single case reports are exempt from ethical approval in our institution, as the paper does not contain any information that identifies the patient. Moreover, consent was taken from the patient.

## Funding

None.

## Author contribution

Ahmad Muhammad: Writing original draft, review and editing, visualization, literature review, and the corresponding author who submitted the paper for publication.

Ahmad Rami Alhayek: writing, conceptualization, and reviewing.

Ahmad Abd Alhai Rehawi: Participated in the operation, conceptualization, and reviewing.

Yousef Hanna: Performed and supervised the operation, conceptualization, and reviewing.

## Guarantor

Ahmad Muhammad.

## Research registration number

N/A.

## Conflict of interest statement

Authors declare that there is no conflict of interest.
